# Croup and Its Management

**Published:** 1888-07

**Authors:** 


Croup and its Management. By Thomas Nichol, M. D., LL.D., D. C. L. Montreal: W. Drysdale & Co., 1887.

Dr. Nichol seems to have undertaken the herculean task of enlightening the good people of the Dominion upon Homœopathy; in so doing he is issuing a series of "Montreal Tracts." This tract upon croup is No. 4 in this series, and purposes to show the much greater curative success of homœopathic therapeutics over those of the old school.

